# Cardiac magnetic resonance imaging for preprocedural planning of percutaneous left atrial appendage closure

**DOI:** 10.3389/fcvm.2023.1132626

**Published:** 2023-06-23

**Authors:** Dagmar Bertsche, Patrick Metze, Erfei Luo, Tillman Dahme, Birgid Gonska, Wolfgang Rottbauer, Ina Vernikouskaya, Volker Rasche, Leonhard M. Schneider

**Affiliations:** Department of Internal Medicine II, Ulm University Medical Center, Ulm, Germany

**Keywords:** left atrial appendage closure, cardiac magnetic resonance imaging, preprocedural planning, landing zone dimensions, angulation prediction

## Abstract

**Introduction:**

Percutaneous closure of the left atrial appendage (LAA) facilitates stroke prevention in patients with atrial fibrillation. Optimal device selection and positioning are often challenging due to highly variable LAA shape and dimension and thus require accurate assessment of the respective anatomy. Transesophageal echocardiography (TEE) and x-ray fluoroscopy (XR) represent the gold standard imaging techniques. However, device underestimation has frequently been observed. Assessment based on 3-dimensional computer tomography (CTA) has been reported as more accurate but increases radiation and contrast agent burden. In this study, the use of non-contrast-enhanced cardiac magnetic resonance imaging (CMR) to support preprocedural planning for LAA closure (LAAc) was investigated.

**Methods:**

CMR was performed in thirteen patients prior to LAAc. Based on the 3-dimensional CMR image data, the dimensions of the LAA were quantified and optimal C-arm angulations were determined and compared to periprocedural data. Quantitative figures used for evaluation of the technique comprised the maximum diameter, the diameter derived from perimeter and the area of the landing zone of the LAA.

**Results:**

Perimeter- and area-based diameters derived from preprocedural CMR showed excellent congruency compared to those measured periprocedurally by XR, whereas the respective maximum diameter resulted in significant overestimation (*p* < 0.05). Compared to TEE assessment, CMR-derived diameters resulted in significantly larger dimensions (*p* < 0.05). The deviation of the maximum diameter to the diameters measured by XR and TEE correlated well with the ovality of the LAA. C-arm angulations used during the procedures were in agreement with those determined by CMR in case of circular LAA.

**Discussion:**

This small pilot study demonstrates the potential of non-contrast-enhanced CMR to support preprocedural planning of LAAc. Diameter measurements based on LAA area and perimeter correlated well with the actual device selection parameters. CMR-derived determination of landing zones facilitated accurate C-arm angulation for optimal device positioning.

## 1. Introduction

Atrial fibrillation (AF) is a major cardiac arrhythmia and is known to increase the risk of mortality (1) including an increased risk of stroke (2, 3). In patients with AF, the left atrial appendage (LAA) was identified as a source of thrombus associated with a higher risk of causing stroke (4) with oral anticoagulation representing an effective therapy to reduce the risk of stroke (5). In patients with contraindications to oral anticoagulation (6) closure of the LAA (LAAc) represents an efficient therapy option (7). However, the high variability of LAA shapes and dimensions (8) challenges optimal occluder selection and final implantation (9, 10), indicating the need for accurate preprocedural assessment of the LAA.

The gold standard imaging modality for LAA assessment for LAAc is 2D transesophageal echocardiography (TEE), also recommended in conjunction with x-ray fluoroscopy (XR) (11). The selection of device size is based on the maximum diameter obtained from the LAA diameters assessed at the anticipated landing zone of the occluder (12). However, despite the high spatial resolution of both imaging modalities, underestimation of the maximal diameter derived from 2D TEE and XR has been reported, which was attributed to the often oval shape of the LAA and the non-optimal choice of view geometry selected in 2D TEE or projective XR for measurement (13–15).

In general there is a high demand for preprocdural assessment of the LAA geometry for accurate procedure planning. The use of 3D TEE has been shown to be more accurate than those based on 2D TEE (14, 16), although the maximum diameter is still underestimated compared to measurements based on 3D computer angiography (CTA) (17, 18). CTA has been shown to be an appropriate imaging tool for LAAc planning (19). In case of 3D imaging data, device size selection based on perimeter and area measurement derived landing zone diameters have been reported to be more reliable than simply using the maximum diameter (16, 20). In addition to the correct assessment of the diameter, the optimal XR angulations for periprocedural imaging during implantation could be retrieved from the CTA data (21). Even though preprocedural assessment of the LAA based on CTA has the potential to improve the outcome and efficiency of the intervention (22, 23), its application is limited in patients with reduced kidney performance and due to its intrinsic risk of ionizing radiation is under safety/benefit debate for general application in LAAc (24). Here, cardiac magnetic resonance imaging (CMR) may gain interest especially considering the additional excellent soft-tissue contrast, enabling thrombus detection in the LAA (25, 26). However, where there is general consensus that 3D CT is appropriate for LAAc planning, the respective role of 3D CMR for LAAc planning needs further investigation (11).

As further approaches for facilitating improved preprocedural device selection printed 3D models either derived from CTA (27, 28) or CMR data (29) as well as an interactive modelling tool for LAAc planning (30) have been reported.

The objective of this pilot study is to investigate the application of non-contrast-enhanced 3D CMR in preprocedural planning of LAAc. For initial accuracy assessment of CMR, the landing zone diameters were quantified retrospectively at the same LAA location as used for the XR-derived periprocedural measurements. For estimation of the value of CMR for LAAc planning, landing zones were determined prospectively based on CMR and the resulting diameters and optimal C-arm angulations compared to periprocedural data.

## 2. Methods

13 patients (85% male, 76 ± 9 years) with atrial fibrillation (paroxysmal, persistent, long-persistent, permanent) not suited for oral anticoagulation therapy were enrolled in this pilot study [*n* = 10 Watchman FLX™ (Boston Scientific, Massachusetts, USA); *n* = 2 LAmbre™ (Lifetech Scientific, Shenzhen, China); *n* = 1 Amplatzer™Amulet™ (Abbott, Illinois, USA)]. The procedure was performed according to the current clinical recommendations. For device size selection, the LAA of the patients was measured periprocedurally with 2D TEE (EPIQ CVxi, Philips Medical Systems, Best, The Netherlands) at view angles ∼45°, ∼90°, and ∼135°. To approve the TEE measurements, contrast-enhanced XR (Allura Clarity, Philips Medical Systems, Best, The Netherlands) were additionally routinely acquired in right anterior oblique and caudal angulation as recommended (31). If according to the objective impression of the interventional cardiologist the LAA was not well displayed in the XR projections acquired in recommended angulation, additional XR angulations were acquired and analyzed. The maximum diameter measurement of the identified landing zone was considered for the selection of the device size. All patients underwent an CMR examination the day before the actual intervention. The evaluation was conducted in accordance with the ethical guidelines of the 1975 Declaration of Helsinki and was approved by the local ethical committee. Written informed consent was obtained from all individual participants included in the study (drks.de DRKS00015649).

### 2.1. Magnetic resonance imaging of the left atrial appendage

Preprocedural 3D CMR data were acquired with a spatial resolution of 1.3^3^ mm^3^ at 3 T (Achieva 3.0 T, dStream, R5.6, Philips Medical Systems B.V., Best, The Netherlands) with a respiratory navigated mDixon sequence and a non-contrast-enhanced protocol according to Homsi et al. (32). The CMR data were acquired in atrial diastole, preferably at 30%–40% phase of the RR-interval as proposed for measuring the LAA dimensions (33).

### 2.2. Accuracy of 3D-CMR

**Figure 1:**
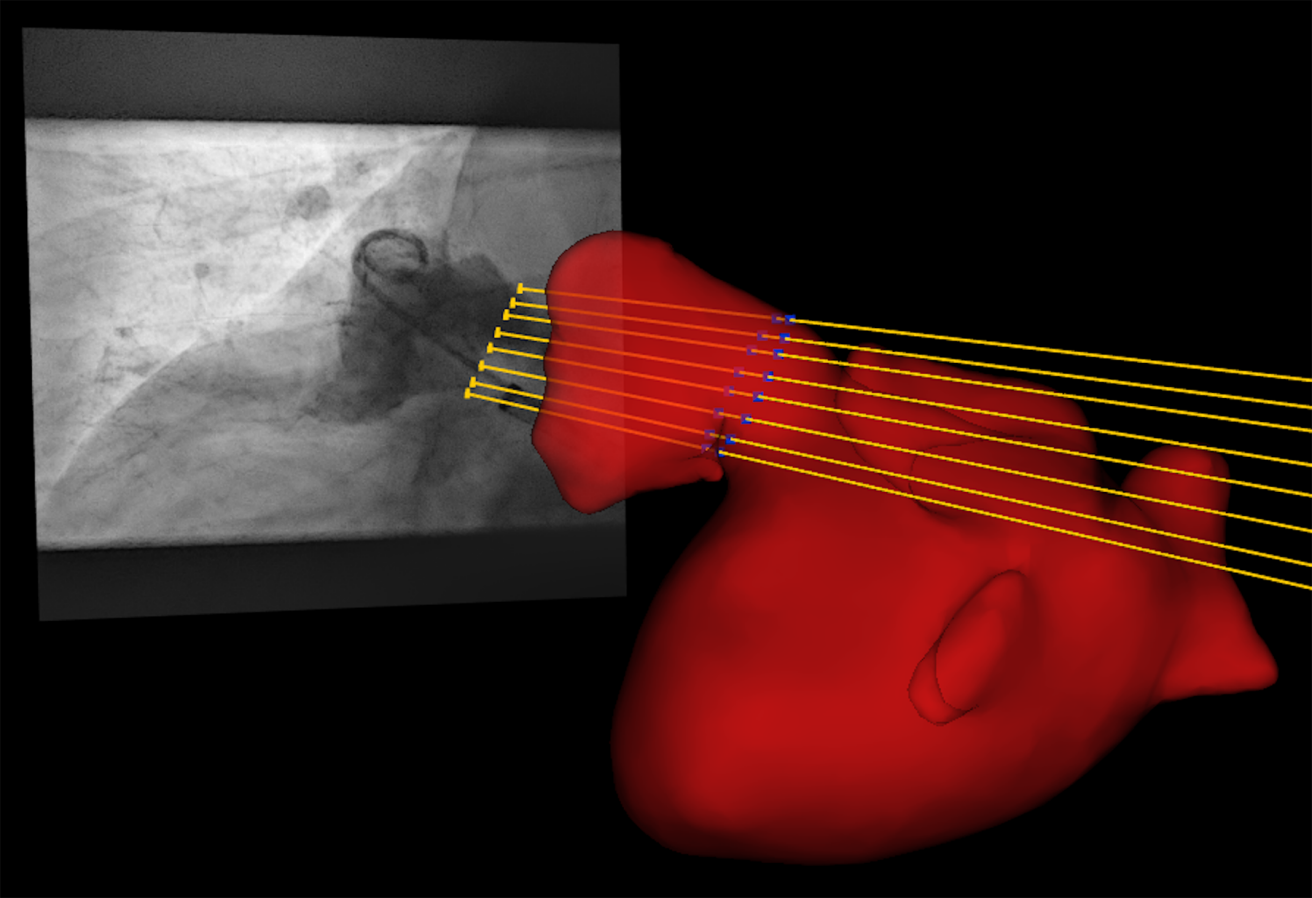
By intersection of the projection lines (yellow lines) of the landing zone identified in XR (yellow dots) with the left atrial appendage segmented from pre-procedural registered CMR (red), the landing zone was localized in 3D (blue dots).

Quantitative comparison of CMR and XR for LAA dimension assessment requires measurement of the respective parameters at the same anatomical location. Since the procedure was performed according to the current clinical recommendations, the respective measurements were done at the location of the landing zone as identified by XR during the LAAc procedure. Accurate identification of the periprocedurally XR-identified landing zone in the CMR data was ensured by registration of the CMR and x-ray data in a common 3D-XR system geometry. According to the geometry values of the XR system stored in a digital imaging and communication in medicine (DICOM) standard image for each XR measurement, each image was visualized geometrically correctly in the 3D XR system geometry. Patient-specific 3D surface meshes were derived from the 3D CMR after manual segmentation of the respective structures using 3DSlicer [www.slicer.org, (34)]. Manual registration of the surface meshes to the XR system geometry was achieved with 3D-XGuide (35). Similar to previous work (36), each XR-derived landing zone was localized in 3D by calculating the intersection points of the projection lines with the 3D surface mesh (Figure 1). A respective 2D CMR image aligned with the landing zone was generated by multi-planar reconstruction (MPR), thus ensuring measurements in the CMR at the same anatomical location as chosen by periprocedural XR.

In the reformatted CMR image the maximum diameter (d’_max_­), the diameter parallel to the actual chosen XR projection plane (d’_proj_), and the minimum diameter (d’_min_) of the XR-derived landing zone were determined. These CMR-based measurements were compared to the periprocedural measurement based on XR (d_XR_). Furthermore, the ovality of the LAA was derived as the difference between d’_min_ and d’_max_ and set into relation to the deviations between d’_max_ and d_XR_.

To assess the interrater reliability of the measurements, data analysis was independently done by three readers for both modalities. Intraclass correlation coefficients (icc) and their 95% confidence intervals (ci) were calculated using the statistical python package pingouin (37) and were rated according to Koo et al. (38). The icc were calculated based on single-rating, absolute-agreement, two-way random-effects models.

### 2.3. Cardiac magnetic resonance imaging for LAAc procedure planning

To investigate the potential of CMR to support preprocedural planning of LAAc, the anticipated landing zones were preprocedurally defined based on the patient's CMR images (3mensio Structural Heart™, V10.2, Pie Medical Imaging, Maastricht, The Netherlands) according to expert recommendations derived from CTA (21). Landing zone diameters and optimal XR angulations were determined and retrospectively compared to clinical data acquired periprocedurally with the clinically recommended procedure.

#### 2.3.1. Landing zone dimensions

**Figure 2:**
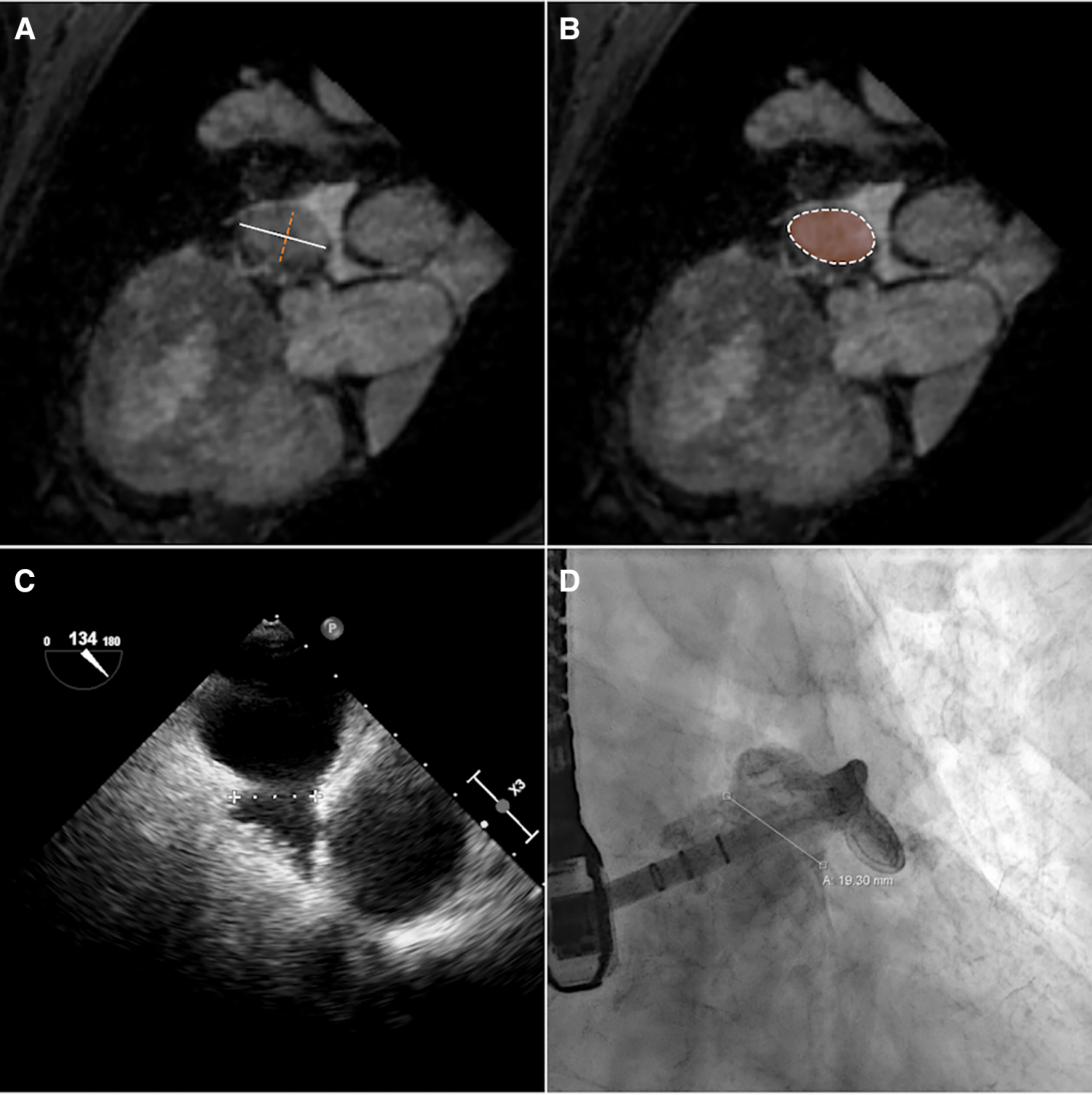
The diameter of the landing zone measured by CMR (**A,B**), TEE (**C**), and XR (**D**). Based on CMR, maximum (**A**, white solid line) and minimum (**A**, orange dashed line) diameters and diameters calculated from perimeter (**B**, white dashed line) and area (**B**, orange area) measurements of the landing zone were derived.

The maximum diameter (d_max_), the perimeter (*p*) derived diameter (d_peri _= $\frac{\;p}{\pi }$), the area (*a*) derived diameter (d_area_ = $2\sqrt{\frac{a}{\pi }}$), and minimum diameter (d_min_) of the CMR-derived landing zone were quantified in the CMR images (Figures 2A,B). CMR-derived measurements were compared with periprocedural measurements derived from XR (d_XR_) and TEE (d_TEE_) (Figures 2C,D). The ovality of the landing zones derived from d_max_ and d_min_ was correlated with the deviations of d_max_ to d_XR_ and d_TEE_.

#### 2.3.2. Angulation prediction

**Figure 3:**
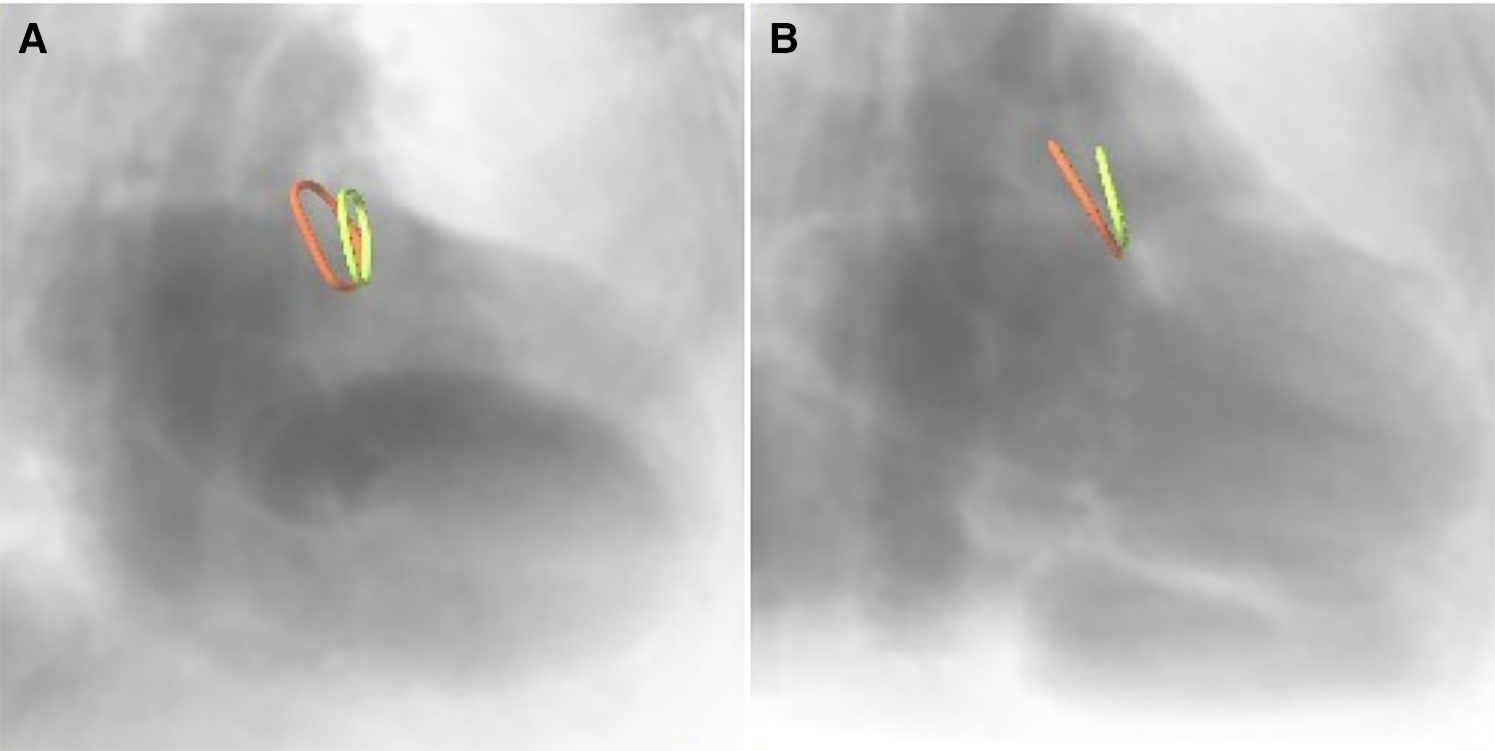
CMR-derived ostium (red) and landing zone (green) superimposed onto angiographical simulation in (**A**) the recommended angulation and (**B**) patient-specific optimal angulation.

Furthermore, the optimal XR angulations were predicted from the CMR identified landing zones yielding orthogonal projection of the maximal diameter (Figure 3) independently by two readers. Angulations deviating by more than the 95% confidence interval were considered different to identify optimal angulations outside of the recommended angulation range (RAO20-30/CAUD20-30).

### 2.4. Statistical analysis

The agreement between the modalities was visually analyzed based on Bland-Altman and scatter plots. The significance of the differences was assessed by applying a paired *t*-test or Wilcoxon signed-rank test as adequate according to Leven's test for equal variances and Shapiro-Wilk test for normality using the python package scipy.stats (39). The correlation was assessed using the Pearson correlation coefficient (*r*) rated according to Taylor (40). A *p*-value <0.05 was assumed statistically significant. The mean value (*m*) and the standard deviation (±, std) of the differences are reported.

## 3. Results

CMR and LAAc were successful in all patients. Two cases (Watchman FLX™) were excluded from the XR-CMR comparison due to the non-availability of suited XR data caused by inadequate XR angulations and final device selection solely based on TEE. In two of the remaining 11 cases initially selected devices were underestimated and required subsequent reselection during the ongoing procedure.

### 3.1. Cardiac magnetic resonance imaging of the left atrial appendage

**Figure 4:**
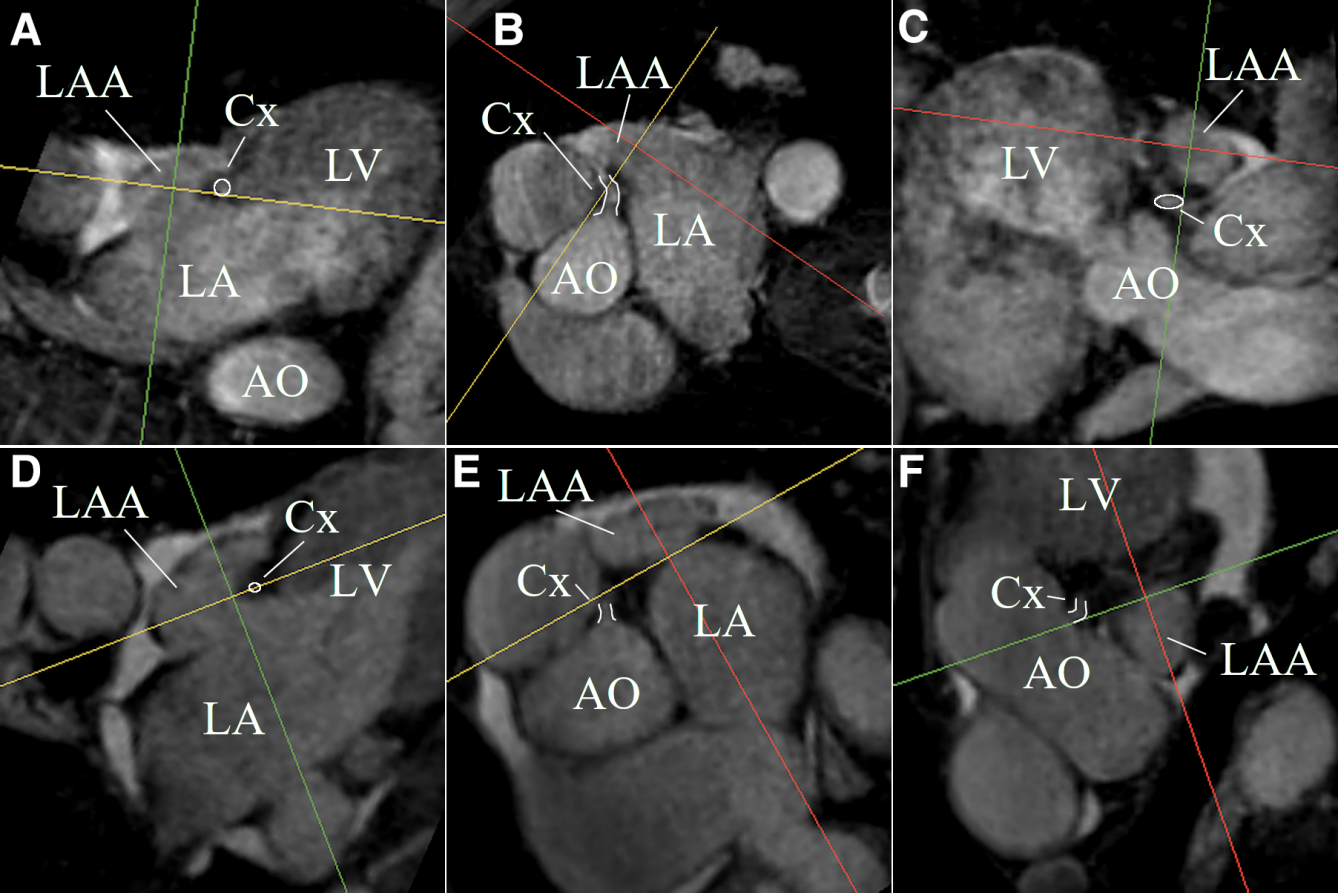
MPR views of two LAA anatomies (first anatomy (**A–C**); second anatomy (**D–F**)) exemplifying the ability of non-contrast-enhanced CMR to identify the LAA anatomy and the landing zone marker Cx (white highlighted).

The LAA could be identified, segmented, and measured on the non-contrast-enhanced CMR despite severe arrhythmia in all patient data sets. Figure 4 shows MPR views of two LAA anatomies exemplifying the ability of non-contrast-enhanced CMR to identify the LAA anatomy and the landing zone marker Cx. The acquisition duration was depending on the heart rate, arrhythmia rejection, and respiration navigator efficiency but could be kept below 15 min in all cases.

### 3.2. Accuracy of 3D-CMR

**Figure 5:**
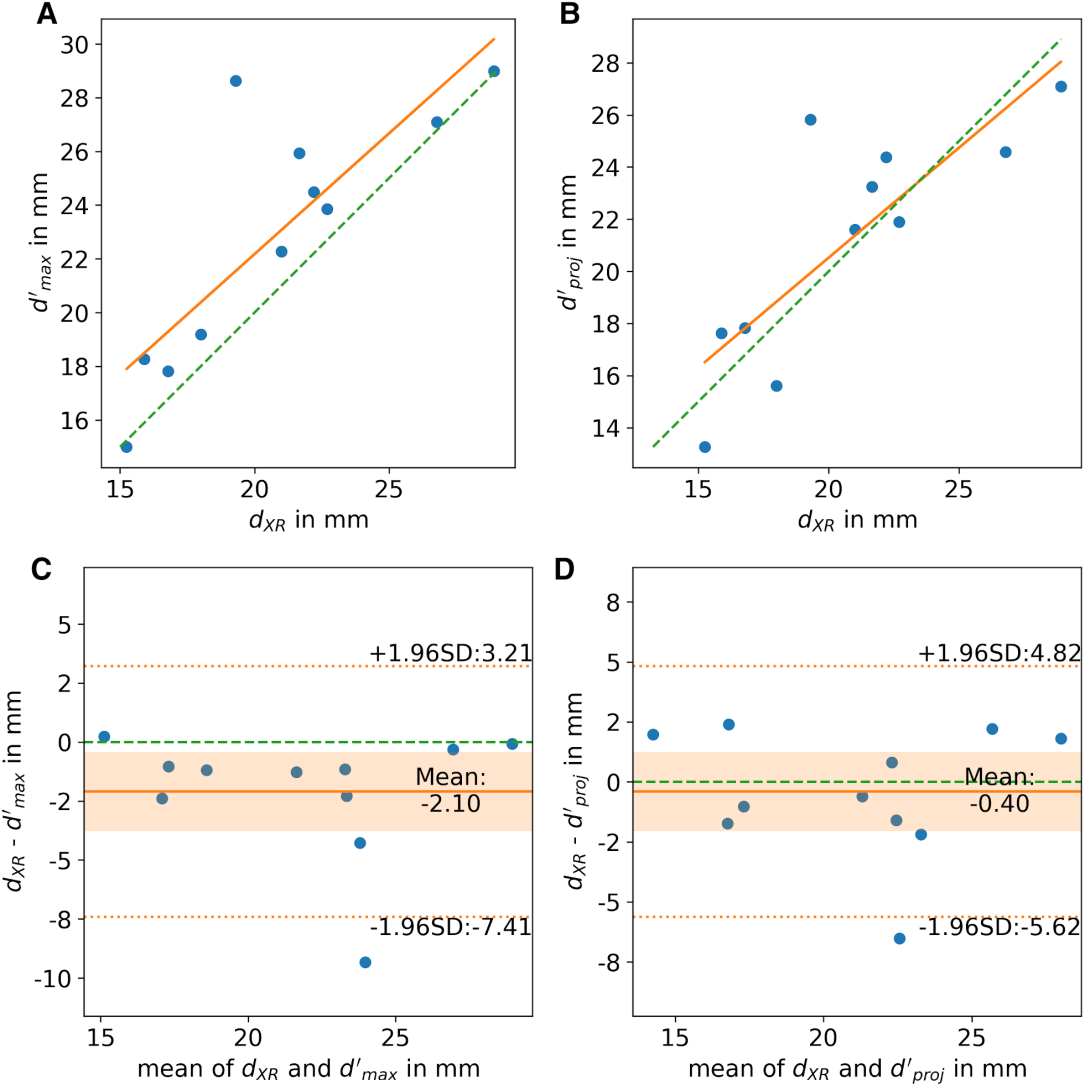
(**A,B**) Correlation and (**C,D**) Bland-Altman analysis of LAA diameters derived from XR (d_XR_) and CMR (d’) imaging at identical landing zone. (**A,C**) Shows the CMR-derived maximum diameter (d’_max_) and (**B,D**) the diameter of the landing zone parallel to the projection plane (d’_proj_). scatter plot: ideal correlation (green dashed line), least square fit through data points (orange line); Bland-Altman plot: zero line (green dashed line), mean difference (orange solid line), limits of agreement (orange dashed line), confidence interval of mean difference (orange area).

In all cases, the landing zone measured in XR angiography could be localized in 3D CMR. The reliability of the XR-based measurements was excellent [icc = 0.95, ci = (0.88, 0.99)]. The reliability of the measurements based on CMR was good for d’_proj_ [icc = 0.91, ci = (0.79, 0.97)], d’_max_ [icc = 0.83, ci = (0.6, 0.94)], and d’_min_ [icc = 0.80, ci = (0.56, 0.93)]. The CMR-based measurements d’_max_ (*r* = 0.83, *p* < 0.05) and d’_proj_ (*r* = 0.82, *p* < 0.05) were strongly correlated with XR-based measurements (Figures 5A,B). For all outliers, observed with >2 mm difference between XR- and MRI-based measurements, the MRI-predicted optimal XR-angulation differed from the classical angulation range. The ovality of the landing zone correlated with the difference of the d_XR_ to d’_max_ (*r* = 0.77, *p* < 0.05). d’_proj_ (*m* = 0.4 ± 2.5 mm, *p* = 0.63) was in good accordance with d_XR_ (Figure 5D). Significant differences were observed for d’_max_ (*m* = 2.1 ± 2.6 mm, *p* < 0.05) with clear overestimation in comparison to the XR measurements (Figure 5C).

### 3.3. Cardiac magnetic resonance imaging for LAAc procedure planning

#### 3.3.1. Dimension assessment

**Figure 6:**
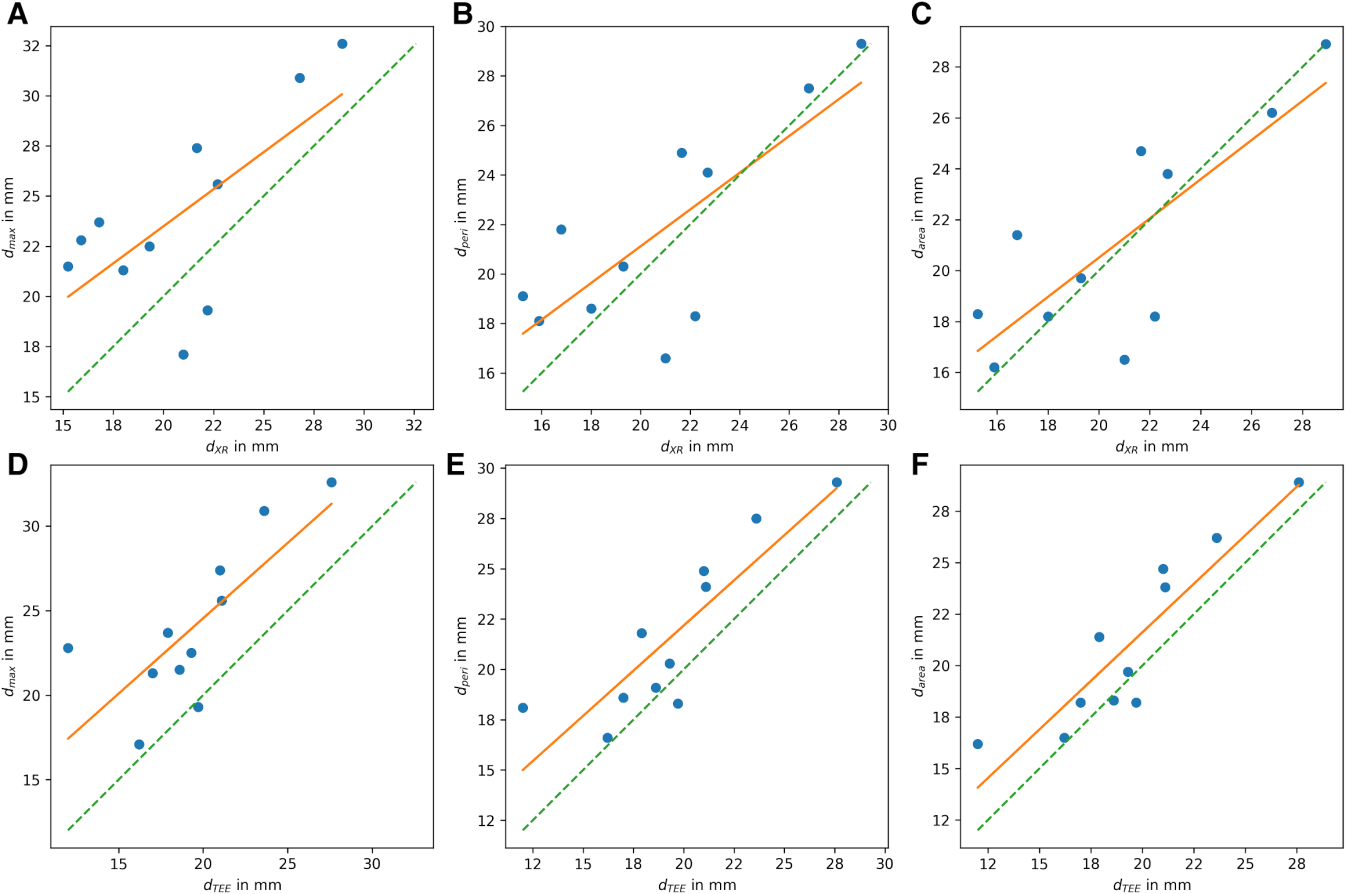
Correlation of LAA diameter at landing zone derived from CMR with (**A–C**) diameters derived from XR (d_XR_) and with (**D–F**) diameters derived from TEE (d_TEE_). (**A,D**) Shows the maximum diameter d_max_, (**B,E**) diameter derived from perimeter (d_peri_), and (**C,F**) diameter derived from area (d_area_) (ideal correleation: green dashed line; least square fit through data points: orange line).

**Figure 7:**
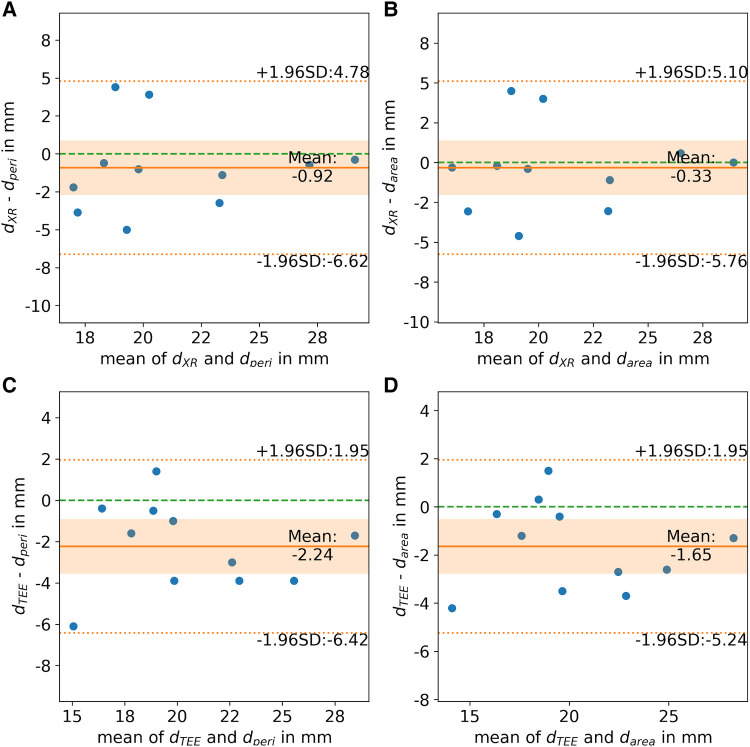
Bland-Altman analysis of landing zone diameters derived from CMR-based landing zone ((**A, C**) perimeter-derived (d_peri_) and (**B, D**) area-derived (d_area_)) in comparison to diameters derived from (**A,B**) x-ray fluoroscopy (d_XR_) and (**C,D**) transesophageal echocardiography (d_TEE_). (Zero line: green dashed line; mean difference: orange solid line; limits of agreement: orange dashed line; confidence interval of mean difference: orange area).

All CMR-based measurements, d_max_ (*r* = 0.68, *p* < 0.05), d_peri_ (*r* = 0.77, *p* < 0.05), and d_area_ (*r* = 0.79, *p* < 0.05), had high correlation with XR-based measurements (Figures 6A–C). Similar to the CMR-based measurements at the landing zone locations derived from XR (d’), d_max_ significantly differed from the XR-based measurements (*m* = 3.3 ± 3.5 mm, *p* < 0.05), while d_peri_ (*m* = 0.9 ± 2.8 mm, *p* = 0.32) and d_area_ (*m* = 0.3 ± 2.6 mm, *p* = 0.70) did not significantly deviate from d_XR_. All CMR-based measurements, d_max_ (*r* = 0.77, *p* < 0.05; *m* = 4.6 ± 2.9 mm, *p* < 0.05), d_peri_ (*r* = 0.87, *p* < 0.05; *m* = 2.2 ± 2.0 mm, *p* < 0.05), and d_area_ (*r* = 0.90, *p* < 0.05; *m* = 1.6 ± 1.7 mm, *p* < 0.05), had high to very high correlation with TEE-based measurements but revealed a significant overestimation (Figures 6D–F). The Bland-Altman analysis reveals a still rather large confidence interval for all assessed parameters (Figure 7). The ovality of the landing zones derived from CMR correlated with the deviation of d_max_ to both, d_XR_ (*r* = 0.73, *p* < 0.05) and d_TEE_ (*r* = 0.84, *p* < 0.05). The statistical analysis values are summarized in Table 1.

**Table 1:** Comparison of LAA diameters periprocedurally derived from XR (d_XR_) and TEE (d_TEE_) with respective diameters prospectively derived from CMR (maximum: d_max_; perimeter-derived: d_peri_; area-derived: d_area_).

	Mean deviation ± standard deviation in mm; *p*-value	Pearson correlation coefficient; *p*-value
d_max_ vs. d_XR_	3.3 ± 3.5; <0.05	0.68; <0.05
d_peri_ vs. d_XR_	0.9 ± 2.8; 0.32	0.77; <0.05
d_area_ vs. d_XR_	0.3 ± 2.6; 0.70	0.79; <0.05
d_max_ vs. d_TEE_	4.6 ± 2.9; <0.05	0.77; <0.05
d_peri_ vs. d_TEE_	2.2 ± 2.0; <0.05	0.87; <0.05
d_area_ vs. d_TEE_	1.6 ± 1.7; <0.05	0.90; <0.05

#### 3.3.2. Angulation prediction

The reliability determinations of optimal angulation prediction on average varied by 7.6°  ± 4.2° with a 95% confidence interval of ci = (5.3°, 9.9°) between the independent reader. This is in good accordance with ranges reported for transcatheter aortic valve interventions (41, 42). Therefore, deviations in the angulation between XR an CMR predictions >10° were considered different.

CMR predicted and periprocedurally used angulations were in good accordance for about 85% of cases (Table 2). The angulation predicted by CMR and periprocedurally used were in the recommended range in 54% of cases. In 31% of cases XR angulation during the procedure had to be adjusted to an angulation outside the recommended range for accurately displaying the LAA, what was predicted by CMR. Of note, in one case reselection of the device was required after initial underestimation in the recommended angulation. In one case (8%) CMR predicted an optimal angulation in the recommended range which was not used during the procedure and in one case (8%) the recommended angulation range was used during the procedure but predicted differently by CMR.

**Table 2:** Confusion matrix CMR proposed and clinically chosen C-arm angulation.

Clinically chosen	CMR-derived angulation prediction	
	Recommended	Different >10°
Recommended	54%	8%
Different >10°	8%	31%

A distinction was made between the clinically recommended angulation range (RAO20-30/CAUD20-30) and angulations differing from this range by more than 10°.

## 4. Discussion

The potential advantages of preprocedural planning for LAAc like improved device size selection and angulation prediction have already been reported for CTA (17, 43). By utilizing a non-contrast-enhanced CMR protocol for preprocedural planning, radiation and contrast agent dose might further be reduced in comparison to CTA. This small pilot study showed the possibility of 3D imaging of the LAA of patients with atrial fibrillation using CMR for preprocedural planning.

Dimension assessment of the LAA based on the CMR at the identical landing zone as the XR-derived measurements showed high congruency indicating potential support of CMR for preprocedural planning. The significant differences in maximal diameters between CMR and XR are likely caused by the limited spatial resolution in CMR and non-optimal XR angulation as also previously reported in the context of preprocedural planning for LAAc based on CTA (13, 23, 44). This assumption was supported by the correlation of the ovality of the LAA with the deviation between the two modalities. Calculation of the CMR derived diameter at similar angulation direction as the XR clearly improved the agreement between the modalities.

From the current data, using the derived diameters for device selection appears feasible, as already motivated for CTA-based measurements (20, 45) and 3D TEE (16). Alternatively, a new device selection chart might be established for measurements based on 3D imaging as the currently used sizing charts are intended for TEE-based measurements and are intrinsically considering the associated underestimation (20). Even though underestimation with TEE has been considered in the device sizing chart, a more accurate LAA size measurement with CT has reported to lead to a lower number of re-device selection (46). LAA dimensions of CMR-derived landing zones overestimated the dimensions derived from TEE significantly, which is in concordance with previous studies (14, 47).

Previous studies (26, 28), as well as this study, compare periprocedural TEE and fluoroscopy measurements with measurements based on preprocedural data. Differences in observed diameters may be explained by the moment of imaging rather than the imaging modality. Patients undergoing TEE during the procedure generally have been fasting for a while, resulting in a more hypovolemic state. This could lead to underestimation of LAA diameter in XR and TEE imaging. This is in line with the current study, showing larger LAA diameters in CMR compared to XR and TEE. Ideally, imaging modalities should be compared at the same moment to account for this possible bias.

A general limitation in CMR rises from the rather low spatial resolution, which did not allow for clear identification of the trabecular structures in the LAA, and hence a clear landmark for exact determination of the landing zone maybe missing. The difficult landing zone identification in CMR may explain the somehow larger deviations of the CMR-derived measurements for device size prediction as compared to the CMR validation at identical landing zones.

However, even with the potentially non-optimal landing zone derived from CMR successful angulation prediction could be performed. The predicted angulation had high congruency with the final angulation clinically chosen showing the high potential of preprocedural CMR-based angulation determination in saving radiation, contrast agent, and interventional time.

A further limitation for CMR in LAAc procedure planning may rise from the rather long scan time, high related costs, and necessary exclusion of patients with non-MR compatible pacemaker. As such, CMR is unlikely to completely replace CT as preprocedural routine imaging modality. Nonetheless, for patients not suitable for CT, e.g., with impaired renal function, CMR has the great potential to become an alternative non-invasive contrast agent-free imaging modality for providing the information required for preprocedural planning. For assessment of the cost effectiveness of this approach larger blinded studies including the assessment of procedure time, procedural success and long-term patient outcome need to be performed.

### 4.1. Perspectives

The results presented here are based on a small number of single-center cases and should be further investigated on a larger scale. Additionally, the investigated TEE-derived LAA measurements are limited to views acquired at 45°, 90°, and 135°. Including measurement at 0° might further improve the accordance between the TEE- and CMR-derived diameters. Best to our knowledge there is currently no planning software for CMR based planning for LAAc approved as a medical device. Besides the prediction of the XR angulation, the prediction of the optimal TEE angulation could also be investigated as the ovality of the landing zone determined in CMR correlates with the deviation of the maximum MRI-based diameter and the diameters measured in XR and TEE. Furthermore, as it appears that CT- and CMR-based measurements of the LAA are larger compared to TEE- and XR-based measurements, a direct comparison of CT and CMR would be valuable to evaluate the respective advantages and disadvantages in the application of LAAc planning in preprocedural tomographic 3D images. Furthermore, the value of CMR predicting optimal transseptal puncture site for an optimal access to the LAA as well as the support of CMR-based periprocedural image fusion should be investigated as already proposed for CT (46) during LAAc.

## 5. Conclusions

This pilot study demonstrates the high potential of non-invasive, contrast agent-, and radiation-free CMR imaging in planning for LAAc. Based on the 3D CMR, the shape of the LAA can be evaluated and the landing zone can be determined for dimension assessment and angulation prediction. However, device size selection based on CMR-derived measurements maybe limited by the current sizing charts. Due to the limited accuracy in the maximal diameter the usage of derived diameters seems to be recommended and investigated in future studies.

## Data Availability

The data analyzed in this study is subject to the following licenses/restrictions: DS-GVO. Requests to access these datasets should be directed to volker.rasche@uni-ulm.de.
